# The serum fascin-1 and tumor components containing this protein in patients with head and neck squamous cell carcinoma: A pilot study

**DOI:** 10.7555/JBR.38.20240397

**Published:** 2025-06-25

**Authors:** Gelena V. Kakurina, Elena E. Sereda, Marina N. Stakheeva, Irina V. Kondakova, Evgeny L. Choinzonov

**Affiliations:** 1 Cancer Research Institute, Tomsk National Research Medical Center, Russian Academy of Sciences, Tomsk, Tomsk region 634009, Russia; 2 Department of Biochemistry and Molecular Biology, Faculty of Medicine and Biology, Siberian State Medical University, Tomsk, Tomsk region 634050, Russia

Dear Editor,

Local recurrence and cervical lymph node metastases are major causes of mortality in patients with head and neck squamous cell carcinoma (HNSCC). To date, none of the proposed strategies for predicting outcomes in this disease have proven fully effective, and a comprehensive physical examination remains the primary method for early detection and monitoring of HNSCC. Therefore, identifying new molecular markers for HNSCC prognosis would be highly beneficial. Candidate biomarkers of HNSCC progression include various signaling proteins and actin-binding proteins (ABPs)^[1]^. ABPs are involved in cytoskeletal rearrangement, facilitating cell motility and contributing to the release of tumor cells into the bloodstream, leading to circulating tumor cells (CTCs). The presence of CTCs is strongly associated with the development of metastases and relapses^[2–3]^, and their determination is approved for predicting outcomes in breast, prostate, and colorectal cancers^[4]^. However, there are morphofunctional and molecular differences between CTCs and primary tumor cells^[3]^. Investigating the molecular characteristics of CTCs, including changes in the composition and expression of ABPs, will help identify new prognostic markers for HNSCC and potential targets for anticancer therapy. Additionally, a comparative analysis of the molecular architecture of CTCs and primary tumor cells will enhance our understanding of tumor progression mechanisms, including those in HNSCC. Based on the significant role of fascin-1 (FSCN1) in the pathogenesis of HNSCC^[1]^ and our previous findings^[5]^, we aimed to study FSCN1 levels in blood serum, peripheral blood leukocytes, and CTCs, as well as FSCN1 expression in tumor cells and in cells of the microenvironment at local sites in HNSCC patients. We also compared one-year treatment outcomes in HNSCC patients based on FSCN1 expression in tumor tissues and its concentration in blood serum.

The current study included 29 Caucasian patients with T1–4N0–1M0 HNSCC, of whom five were female. The median age of the patients was 56 years (range, 43–67). Among these patients, 37.9% (*n* = 11) had regional lymph node metastases (T2–4N1–2М0). Histological examination revealed squamous cell carcinoma with varying differentiation grades in all cases. Blood serum and tumor tissues were collected from patients treated at the Cancer Research Institute (Tomsk, Russia).

The serum level of FSCN1 was measured using an ELISA Kit for FSCN1 (Cloud-Clone Corp., Wuhan, China) and a Multiskan FC enzyme immunoassay analyzer (Thermo Scientific, Shanghai, China). The number of leukocytes and CTCs in whole blood samples was assessed by flow cytometry using a BD FACSCanto Ⅱ flow cytometer (BD Biosciences, Franklin Lakes, NJ, USA). Membrane markers CD45 and CD326 (also known as EpCAM) were stained using Alexa Fluor® 700 mouse anti-human CD45 (monoclonal, clone HI30) and PerCP-Cy™5.5 mouse anti-human EpCAM (monoclonal, clone EBA-1; BD Pharmingen™, San Diego, CA, USA), respectively. FSCN1 was stained after pre-permeabilization of the cell membrane using a Transcription Factor Buffer Set (BD Pharmingen) and a rabbit polyclonal antibody against fascin (phospho-Ser39) conjugated to PE (Biorbyt Ltd., Cambridge, UK). FSCN1^+^ cell subpopulations were sequentially isolated from this gate and expressed as a percentage of the total CTC pool.

Ultrathin sections (7 µm) of tumor tissue were prepared for immunofluorescence analysis using a Leica RM2255 microtome (Leica Biosystems, Newcastle upon Tyne, UK). Staining was performed using a Bond RXm immunostainer (Leica Biosystems). Nuclei in sections were counterstained with DAPI (Leica Biosystems). The panels of marker antibodies (Thermo Fisher Scientific, Waltham, MA, USA) and Opal fluorochromes (Akoya Biosciences, Marlborough, MA, USA) used included: anti-fascin, polyclonal (570; 620); anti-CD20, polyclonal (650); anti-CD68 (clone KP1; 690); and anti-smooth muscle actin (clone 1A4; 690) (***Supplementary Tables 1*** and ***2***, available online). The images were obtained using a Vectra 3.0.3 multiplex tissue analysis system (PerkinElmer, Hopkinton, MA, USA) and processed with inForm 2.2.1 software (PerkinElmer) and spectral libraries. The percentage of tumor cells expressing the target protein was estimated. Data analysis was conducted using SPSS Statistics software, version 22.0 (IBM Corp., Armonk, NY, USA; ***Supplementary Methods***, available online).

In HNSCC patients with lymph node metastases (T2–4N1M0), serum levels of FSCN1 were 10-fold higher than in patients without clinically confirmed regional lymph node metastases (T1–4N0M0; *P* < 0.05; ***Fig. 1A***). Additionally, in patients with disease progression within 12 months following treatment, pre-treatment serum FSCN1 levels were higher than in those without disease progression (*Р* = 0.03; ***Fig. 1B***).

**Figure 1:**
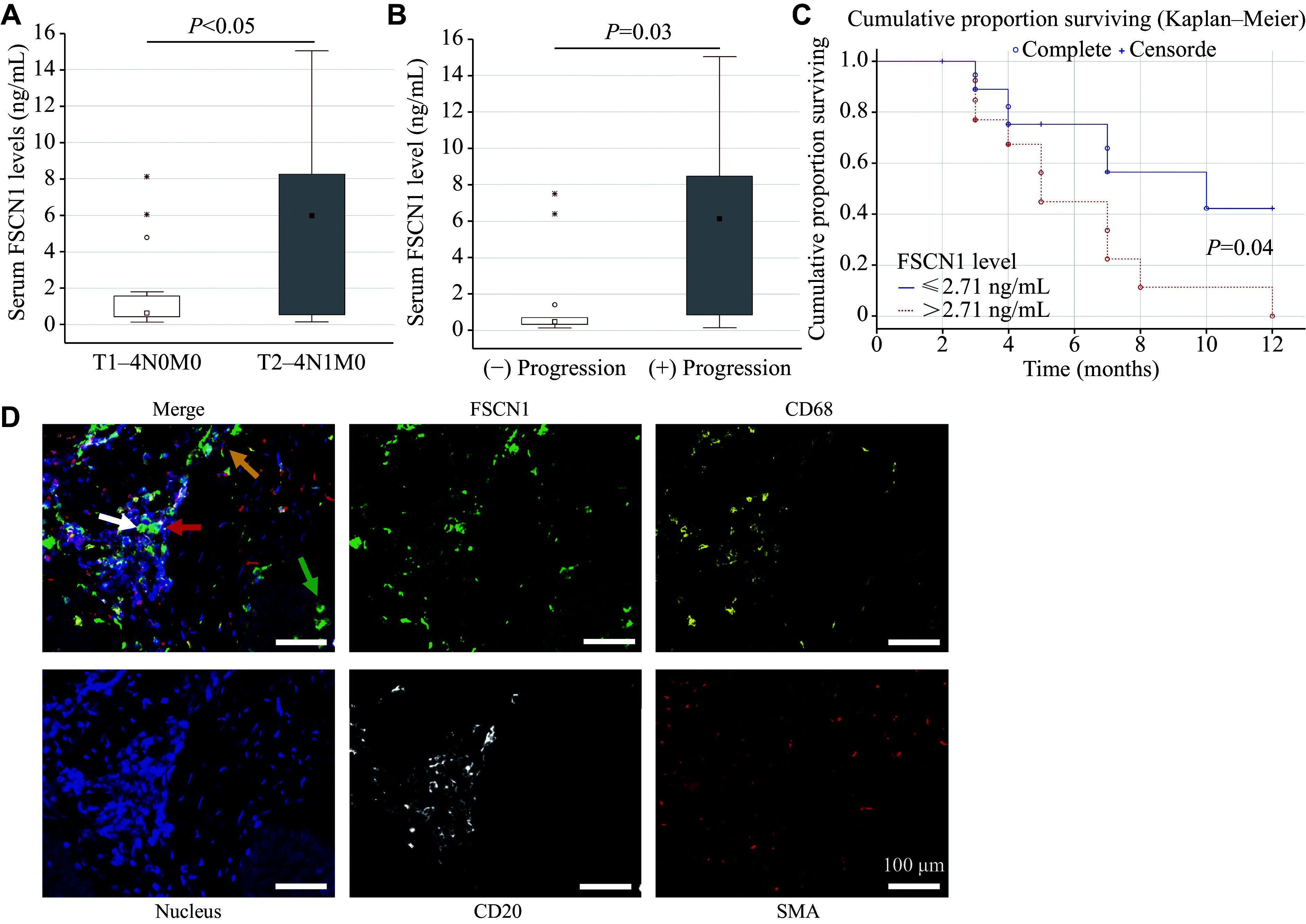
Expression of FSCN1 in HNSCC patient samples. A and B: Depending on the presence of lymph node metastases (A) and tumor progression (B) after anticancer treatment. C: Kaplan–Meier analysis of the 1-year progression-free survival in HNSCC patients according to the serum level of FSCN1. *P* < 0.05 by the log-rank test. D: Expression of FSCN1 in tumor cells (green arrow), macrophages (yellow arrow), B-lymphocytes (white arrow), and fibroblasts (red arrow) infiltrating the stroma of laryngeal squamous cell carcinoma. Multiplex TSA-associated staining. Magnification ×200. Abbreviations: T1–4N0M0, patients without lymph node metastases; T2–4N1–2M0, patients with lymph node metastases; "(−) progression", patients without progression; "(+) progression", patients with progression; CD68, marker of tumor-associated macrophages; CD20, marker of pan-B cells; SMA, marker of activated fibroblasts.

***Fig. 1D*** demonstrates the staining results of tissue sections from squamous cell carcinoma of the larynx. In the upper left square, arrows indicate tumor cells containing FSCN1. Differences in the levels of FSCN1^+^ tumor cells, FSCN1^+^ tumor-associated macrophages, and fibroblasts in tumor tissues were observed with respect to lymph node metastases and tumor progression (***Table 1***). The results showed that in tumor tissues of HNSCC patients, the FSCN1 levels were significantly higher in tumor cells and B-lymphocytes than in macrophages and fibroblasts. In patients with disease progression, the proportion of FSCN1^+^ tumor cells was six-fold higher than in patients without progression (*Р* ˂ 0.05).

**Table 1:** The levels of FSCN1-containing cells in the tissue and peripheral blood of HNSCC patients with respect to the main clinical and morphological characteristics

FSCN1^+^	T1–4N0M0	T2–4N1–2M0	*P* ^a^	(–) Progression	(+) Progression	*P* ^b^
Tumor cells	2.36 (0.19, 17.04)	5.02 (1.64, 17.98)	0.08	1.65 (0.19, 3.09)	10.65 (1.10, 18.12)	˂0.05
B-lymphocytes	4.98 (2.74, 6.89)	6.19 (5.20, 8.38)	0.11	6.17 (3.98, 8.28)	5.20 (3.76, 6.66)	0.51
Macrophages	1.55 (0.81, 2.98)	0.61 (0.13, 2.98)	0.34	1.73 (0.85, 2.79)	0.87 (0.29, 3.17)	0.54
Fibroblasts	0.43 (0.00, 2.85)	0.36 (0.00, 1.54)	0.45	0.09 (0.00, 0.88)	0.97 (0.02, 2.24)	0.06
СD45^+^ leukocyte	34.00 (27.80, 60.70)	44.82 (31.50, 75.70)	0.51	–	–	–
CD326^+^ CTCs	96.9 (90.00, 100.00)	89.10 (84.60, 90.43)	0.05	–	–	–
*Р* ^c^	˂0.05	0.32		–	–	–
The number of cells containing FSCN1 is represented as a fraction of the total population of the respective cells (%). T1–4N0M0, patients without lymph node metastases; T2–4N1–2M0, patients with lymph node metastases; "(–) progression", patients without progression; "(+) progression", patients with progression. The parameters were analyzed using the Mann-Whitney *U*-test.^a^*P*-values indicating significance of differences between the T1–4N0M0 and the T2–4N1–2M0 groups.^b^*P*-values indicating significance of differences between patients without progression and patients with progression.^c^*P*-values indicating significance of differences between FSCN1^+^CD45^–^CD326^+^ and FSCN1^+^CD45^+^ cells.						

The number of FSCN1^+^ CD45^−^CD326^+^ CTCs was approximately threefold higher than that of FSCN1^+^ CD45^+^ cells within the total leukocyte pool (***Table 1***). The proportion of FSCN1^+^ tumor cells in peripheral blood samples (median, 90.9%; interquartile range [IQR], 85.4%–100.0%) was significantly higher than in tissue samples (median, 6.09%; IQR, 1.10%–18.25%) (***Supplementary Fig. 1A*** [available online]). Additionally, positive correlations were found between the proportion of FSCN1^+^CD326^+^ CTCs and tumor size [T(1–4)] (*r* = 0.8; *P* = 0.03), as well as between the proportion of FSCN1^+^ tumor cells and FSCN1^+^ fibroblasts in tumor tissue (*r* = 0.9; *P* = 0.02). A negative correlation between the proportion of FSCN1^+^CD326^+^ CTCs and FSCN1^+^CD45^+^ leukocytes (*r* = −0.7; *P* = 0.03) was also observed.

Our findings suggest that serum levels of FSCN1 may serve as a predictor of lymph node metastasis (AUC = 0.72, 95% confidence interval [CI]: 0.57–0.85; 75% sensitivity and 67% specificity) and progression-free survival (AUC = 0.76, 95% CI: 0.58–0.90; 71% sensitivity and 80% specificity) in HNSCC patients, respectively (***Supplementary Fig. 1B*** and ***1C ***[available online]). Disease progression within 12 months after anticancer treatment was observed in 54.5% of HNSCC patients. Using a cutoff value of 2.71 ng/mL for serum FSCN1 levels to stratify patients with high and low FSCN1 expression, we observed significant differences in progression-free survival rates between the two groups (***Fig. 1C***).

Currently, FSCN1 is proposed as a marker of metastasis and a potential therapeutic target (https://www.clinicaltrials.gov/study/NCT05023486). However, there are "gaps" in understanding the role of FSCN1 in tumor progression, including in HNSCC. Tumor cells are morphologically and functionally heterogeneous in primary tumors, including HNSCC^[6]^, and the heterogeneity of CTCs complicates their identification^[7]^. Therefore, analyzing the molecular characteristics of tumor cells from both the primary tumor and CTCs, including the composition of ABPs, is crucial. In the current study, we demonstrated the role of FSCN1 in the progression of HNSCC by analyzing serum levels of FSCN1 and estimating the number of cells expressing FSCN1 in the tumor and the number of CTCs containing this protein.

Disease progression in HNSCC patients was accompanied by a significant increase in serum levels of FSCN1, consistent with findings from other studies^[1,5,8]^. Additionally, we found that FSCN1 expression was higher in tumor tissue cells than in cells of the tumor microenvironment, and the relative number of circulating FSCN1^+^ tumor cells was approximately threefold higher than the number of FSCN1^+^ leukocytes. Moreover, the proportion of FSCN1^+^ CTCs was significantly higher in peripheral blood samples than in tumor tissue samples. The high number of FSCN^+^CD326^+^ cells in HNSCC may be associated with the formation of an immunological synapse between tumor and immune cells^[9]^. This should be considered when developing therapeutic targets or prognostic markers.

Notably, we found that the level of FSCN1^+^ tumor cells in tissue was 6.45-fold higher in HNSCC patients with relapses within one year after anticancer treatment compared with HNSCC patients without disease progression (*P* ˂ 0.05).

A positive relationship between FSCN1^+^ tumor cells in tissue samples and CTCs in the peripheral blood of HNSCC patients was identified. A close relationship between FSCN^+^ tumor cells in the primary tumor tissue and FSCN^+^ tumor fibroblasts suggests complex interactions between the tumor and its microenvironment^[9]^. FSCN1 is reported to play a key role in regulating cell motility and migration to promote tumor progression^[7–8]^. It is also known that tumor cells may recruit immune cells, creating conditions for tumor growth and escape from immune surveillance^[10]^. Cell models have shown that FSCN1 inhibitors can differentially affect immune cells and alter their interactions. The authors note that *in vivo* studies are needed to assess the effects of FSCN1 inhibition on various cells in the tumor microenvironment^[11]^. The current study indicates that, when developing drugs targeting FSCN1 inhibition, it is essential to consider systemic effects that will also affect immune cells in the human body. Additionally, it is necessary to acknowledge that inhibiting one target may activate alternative bypass signaling pathways and potentially allow dormant tumor cells to survive (*e.g.*, *via* Akt/mTOR), leading to tumor progression and drug resistance^[12]^.

In conclusion, to our knowledge, we are the first to provide a comprehensive assessment of the content of FSCN1 in various biological samples of patients with HNSCC. The relative number of FSCN1^+^ tumor cells in tissue samples was shown to correlate with HNSCC progression. The assessment of serum levels of FSCN1 may be promising for predicting the risk of progression within 12 months after treatment in patients with HNSCC. It was also found that the relative number of FSCN1^+^ CTCs in the bloodstream is greater than that of FSCN1^+^ tumor cells in the primary tumor tissue. Thus, our findings provide a deeper understanding of the significance of fascin-1 in the pathological mechanisms of HNSCC progression.

One limitation of the study is the small sample size. This prevented adequate statistical analysis in HNSCC patient groups stratified by pathological differentiation of the tumor (four well-differentiated and 25 moderately differentiated tumors), sex (five female patients), and median age. Additionally, the group without metastases included patients with stages T1N0M0 (*n* = 2) and T4N0M0 (*n* = 3).

Yours sincerely,Gelena V. Kakurina^1,2,^^✉^, Elena E. Sereda^1,2,△^, Marina N. Stakheeva^1,△^, Liubov Tashireva^1,△^, Olga V. Cheremisina^1,△^, Irina V. Kondakova^1^, Evgeny L. Choinzonov^1^ ^1^Cancer Research Institute, Tomsk National Research Medical Center, Russian Academy of Sciences,Tomsk, Tomsk region 634009,Russia;^2^Department of Biochemistry and Molecular Biology, Faculty of Medicine and Biology, Siberian State Medical University,Tomsk, Tomsk region 634050,Russia. ^△^These authors contributed equally to this work.^✉^Corresponding author: Gelena V. Kakurina. E-mail: kakurinagv@oncology.tomsk.ru.

## SUPPLEMENTARY DATA

Supplementary data to this article can be found online.
