# Supplementary material for: The serum fascin-1 and tumor components containing this protein in patients with head and neck squamous cell carcinoma: A pilot study
Source: J Biomed Res. 2025 Jun 25;39(5):534–7. doi: 10.7555/JBR.38.20240397 (PMC12481671; doi:10.7555/JBR.38.20240397)
Supplement: Supplementary file 1 — Supplementary data to this article can be found online. [file jbr-39-5-534-Supplementary.pdf]

Available online at www.jbr-pub.org.cn

Open Access at PubMed Central

JBR

Journal of Biomedical Research, 2025 39(5): 1–2

Supplementary Data

The serum fascin-1 and tumor components containing this protein in patients with head and neck squamous cell carcinoma: A pilot study

Gelena V. Kakurina[1,2,✉], Elena E. Sereda[1,2,△], Marina N. Stakheeva[1,△], Liubov Tashireva[1,△], Olga V. Cheremisina[1,△], Irina V. Kondakova[1], Evgeny L. Choinzonov[1]

[1]*Cancer Research Institute, Tomsk National Research Medical Center, Russian Academy of Sciences, Tomsk, Tomsk region 634009, Russia;*
[2]*Department of Biochemistry and Molecular Biology, Faculty of Medicine and Biology, Siberian State Medical University, Tomsk, Tomsk region 634050, Russia.*

Supplementary methods

TSA-modified tissue immunofluorescence assay

Ultrathin sections (7 μm) of tumor tissue were prepared for immunofluorescent analysis using a Leica RM2255 microtome (Leica Biosystems, Newcastle upon Tyne, UK). Staining was performed using a BondRXm immunostainer (Leica Biosystems). The staining cycle consisted of several stages: unmasking at 98.5 ℃ for 20 min in Bond Retrieval Solution 2 (Leica Biosystems); washing sections at 25 ℃ for 5 min in Bond Wash Solution (Leica Biosystems); application of Peroxidase Block (Leica Biosystems) at 25 ℃ for 10 min; washing in Bond Wash Solution (Leica Biosystems) at 25 ℃ for 5 min; application of Protein Block (Leica Biosystems) at 25 ℃ for 10 min. Sections were then washed in Bond Wash Solution (Leica Biosystems) at 25 ℃ for 5 min. Primary antibodies were then applied for 30 min. Sections were washed and applied with Post Primary (Leica Biosystems) for 30 min at 25 ℃. After washing twice, Novolink Polymer (Leica Biosystems) was applied for 30 min and incubated at 25 ℃. The wash cycle was repeated two times, and Opal fluorochromes (Akoya Biosciences, Marlborough, MA, USA) were then applied at a dilution of 1∶150, followed by washing and unmasking. Staining of nuclei in sections was performed using DAPI (4',6-diamidino-2-phenylindole) (Leica Biosystems). After staining the nuclei, the sections were mounted in Mounting Medium (Dako, USA).

To mathematically exclude autofluorescence, spectral profiles of unstained tissue samples as well as spectral profiles of Opal and DAPI fluorochromes were created to validate the true fluorescence signal of the desired markers. The images were obtained using a Vectra 3.0.3 multiplex tissue analysis system (PerkinElmer, Hopkinton, MA, USA) and processed using inForm 2.2.1 software (PerkinElmer), taking into account spectral libraries. The proportion of cells expressing the target protein from all tumor cells, expressed as a percentage, was estimated.

Statistical analysis

The IBM SPSS Statistics 22.0 software package was used for data analysis. The results presented in the tables were expressed as median [Q1, Q3], where Q1 and Q3 are the lower and upper quartiles. The Shapiro-Wilk test was used to assess whether the data followed a normal distribution. The nonparametric Mann-Whitney *U*-test was used to compare groups,

△These authors contributed equally to this work.

✉Corresponding author: Gelena V. Kakurina, Laboratory of Tumor Biochemistry, Cancer Research Institute, Tomsk National Research Medical Center, Russian Academy of Sciences, 5 Kooperativny Street, Tomsk, Tomsk region 634009, Russia; Department of Biochemistry and Molecular Biology, Faculty of Medicine and Biology, Siberian State Medical University, 2 Moskovsky trakt, Tomsk, Tomsk region 634050, Russia. E-mail: kakurinagv@oncology.tomsk.ru.

Received: 15 November 2024; Revised: 19 June 2025; Accepted: 24 June 2025; Published online: 25 June 2025

CLC number: R739.91, Document code: B

The authors reported no conflict of interests.

This is an open access article under the Creative Commons Attribution (CC BY 4.0) license, which permits others to distribute, remix, adapt and build upon this work, for commercial use, provided the original work is properly cited.

© 2025 by Journal of Biomedical Research.

https://doi.org/10.7555/JBR.38.20240397

and the Spearman rank correlation was used to assess the relationships between variables. Differences were considered significant at $P < 0.05$.

Progression-free survival (PFS) in HNSCC patients was estimated. Calculations were carried out at the 12th month after completing combined modality treatment, taking into account the patients lost to follow-up. Progression-free survival was calculated in months from the completion of treatment to the time of the last visit or follow-up contact. The follow-up time ranged from three to 12 months. The 1-year PFS rate in HNSCC patients was 45.5%, with a median PFS rate of five months. When assessing the relationship between the serum level of fascin-1 and the 1-year PFS rates, the log-rank test was used. Cumulative survival curves were constructed using the Kaplan-Meier method. Sensitivity, specificity, and prognostic significance of candidate markers were determined by receiver operating characteristic analysis according to the rule for calculating areas under the curve. Differences were considered significant at $P < 0.05$.

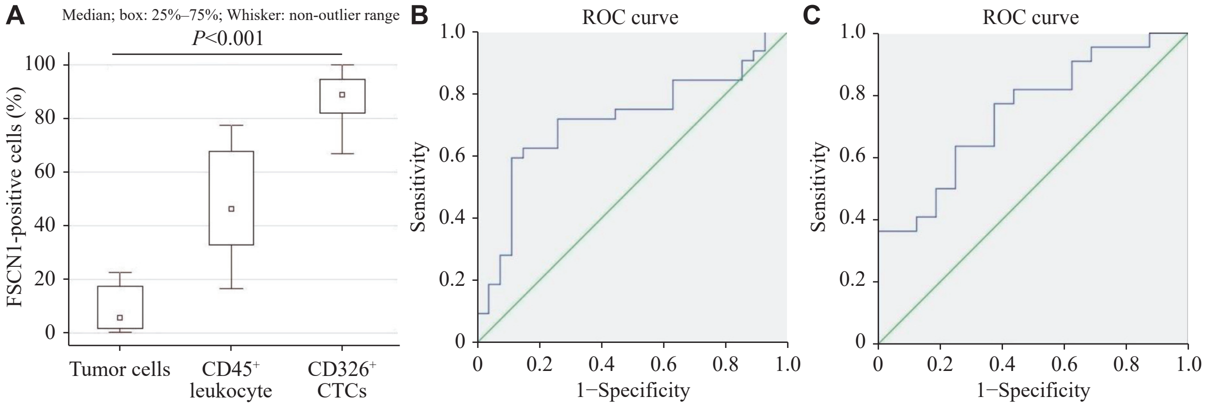

Supplementary Fig. 1 **The number of FSCN1-positive leukocytes, circulating tumor cells in the blood, and tumor cells in tissues (A), as well as the assessment of the sensitivity and specificity of FSCN1 serum levels for predicting lymph node metastases (B) and predicting progression-free survival in patients with HNSCC (C).**

Supplementary Table 1 **The panel No. 1 of markers and Opal fluorochromes for TSA-modified immunofluorescent analysis of tumor tissues in HNSCC patients**

Antibody	Dilution	Manufacturer	Opal	Excitation/Emission
Anti-fascin, polyclonal	1 : 800	Thermo Fisher, USA	570	550 nm/570 nm
Anti-CD20, polyclonal	1 : 400	Thermo Fisher, USA	650	627 nm/650 nm
Anti-CD68, clone KP1	1 : 400	Thermo Fisher, USA	690	676 nm/694 nm

Supplementary Table 2 **The panel No. 2 of markers and Opal fluorochromes for TSA-modified immunofluorescent analysis of tumor tissues in HNSCC patients**

Antibody	Dilution	Manufacturer	Opal	Excitation/Emission
Anti-fascin, polyclonal	1 : 800	Thermo Fisher, USA	620	588 nm/616 nm
Anti-smooth muscle actin, clone 1A4	1 : 500	Thermo Fisher, USA	690	676 nm/694 nm
